# Distinguishing between determinate and indeterminate growth in a long-lived mammal

**DOI:** 10.1186/s12862-015-0487-x

**Published:** 2015-10-14

**Authors:** Hannah S. Mumby, Simon N. Chapman, Jennie A. H. Crawley, Khyne U. Mar, Win Htut, Aung Thura Soe, Htoo Htoo Aung, Virpi Lummaa

**Affiliations:** Department of Animal and Plant Sciences, University of Sheffield, Western Bank, Sheffield, S10 2TN UK; Ministry of Environmental Conservation and Forestry, Myanma Timber Enterprise, Yangon, Myanmar

**Keywords:** *Elephas maximus*, Dimorphism, Life-history evolution, Reproductive strategies, Trade-offs

## Abstract

**Background:**

The growth strategy of a species influences many key aspects of its life-history. Animals can either grow indeterminately (throughout life), or grow determinately, ceasing at maturity. In mammals, continued weight gain after maturity is clearly distinguishable from continued skeletal growth (indeterminate growth). Elephants represent an interesting candidate for studying growth because of their large size, long life and sexual dimorphism. Objective measures of their weight, height and age, however, are rare.

**Results:**

We investigate evidence for indeterminate growth in the Asian elephant *Elephas maximus* using a longitudinal dataset from a semi-captive population. We fit growth curves to weight and height measurements, assess sex differences in growth, and test for indeterminate growth by comparing the asymptotes for height and weight curves. Our results show no evidence for indeterminate growth in the Asian elephant; neither sex increases in height throughout life, with the majority of height growth completed by the age of 15 years in females and 21 years in males. Females show a similar pattern with weight, whereas males continue to gain weight until over age 50. Neither sex shows any declines in weight with age.

**Conclusions:**

These results have implications for understanding mammalian life-history, which could include sex-specific differences in trade-offs between size and reproductive investment.

**Electronic supplementary material:**

The online version of this article (doi:10.1186/s12862-015-0487-x) contains supplementary material, which is available to authorized users.

## Background

Growth strategies are central to our understanding of life-history theory [1], as they determine body size and influence key life-history traits, including survival, development, and reproduction [2, 3]. The life-history trade-off between growth and reproduction enforced by limited energy underlines the importance of resource allocation in the current theory. Notably, this pivotal trade-off occurs against a background of increasing mortality rates with age after maturity [4]. In this scenario, investing resources in reproduction rather than growth represents the most efficient strategy to improve individual fitness. However, the view that this scenario is universal has been challenged by relatively recent concepts such as negative senescence, in which mortality rate declines after reproductive maturity in much the same way as it declines during growth [5]. In this case, investing in continued growth as well as reproduction is in fact the optimal strategy, as the organism can experience the benefits of larger body size alongside improving fitness [3]. Evidence for this strategy has hereto only been found in some corals, plants, molluscs, sea urchins and possibly basal fish [5], which all exhibit growth throughout life. However, a lack of clearly defined characterisation of growth strategies means that there is the potential for negative senescence to exist in a wider range of species.

For iteroparous species, those reproducing more than once, there are two growth strategies available: determinate growth, whereby growth ceases around/slows considerably after sexual maturity [6], and indeterminate growth, which, at the most basic level, involves continued growth through life and is found in the majority of iteroparous animals [7]. However, because indeterminate growth is not consistently defined in the literature and there is a lack of consensus on the definition, species that would otherwise be considered as indeterminately growing may have their growth erroneously labelled as determinate or vice versa. Reptiles are one group particularly affected by this issue [8], caused in part by inadequate existing data. As such, relatively few reptile species can be definitively classified as indeterminate [9, 10], with the majority still unknown or contentious. Even with a conservative definition of indeterminate growth, having too small a sample size in studies on growth can show spurious trends. For example, 20 % of individuals across turtle species do not show indeterminate growth (based on body length measures), even though their species as a whole do [8], meaning that insufficient study sample size could lead to the incorrect conclusion that those turtle species grow determinately.

Misclassification has also occurred amongst higher vertebrates, such as orangutans *Pongo pygmaeus* [11], despite lack of solid evidence of most, if not all, mammals showing any kind of indeterminate growth [7]. Furthermore, northern elephant seals *Mirounga angustirostris* and northern fur seals *Callorhinus ursinus* have been proposed to exhibit indeterminate growth based upon length measurements [12, 13]. These studies remain inconclusive, as they were fully cross-sectional. There may also be seasonal fluctuation in length of seals [12], though the cross-sectional nature of the study cannot distinguish between a true fluctuation or simply variation between cohorts. Although an increasing body weight alone is accepted to represent indeterminate growth amongst soft-bodied invertebrates [6, 14–17], this should not be considered true growth in mammals, and instead represents successful acquisition of resources [18]. Rather, measures of both weight and height/length should be taken, with the latter used as an indicator of skeletal growth and used to distinguish between determinate and indeterminate growth. We use this definition of determinate growth for mammals throughout this paper. Changes in body weight can then be analysed separately. In some cases they might be revealing concerning patterns of resource availability, for example, there is a wealth of literature documenting obesity in zoo primates [19–23]. This highlights an issue with studies on long-lived species – study populations are often captive, and therefore not necessarily representative of the species in the wild; veterinary care can allow weaker individuals to survive, and the provision of food eliminates the need for captive animals to expend energy foraging.

However, obesity may not be the only reason for the continued weight gain observed in species such orangutans, as it could also represent a male life history strategy. Investment in body size by some males after sexual maturity is associated with dominance and access to mates, to the extent that two different male orangutan adult phenotypes exist based on body size [24]. The potential importance of understanding how both growth strategy and adult size dimorphism relate to life history mean that measures of both body height or length (for distinguishing between indeterminate and determinate growth) and weight (for understanding adult body size strategy) provide useful information.

When a growth strategy and its assumptions form the foundations of further theoretical work and conservation efforts [25], incorrectly classifying growth in such a way can potentially impact future life-history research and even the long-term success of a species. Determining how a species grows and when it is likely to reproduce allows more effective management of populations [26–28]. Discerning which growth strategy these potentially misclassified species follow is therefore critical to avoid at best confusion and at worst misdirection. Additionally, indeterminate growth is already thought to drive negative senescence [5] through its association with low adult mortality. If any higher vertebrates exhibit indeterminate growth then it may be the case that they to do not follow the traditionally accepted life-history assumptions either. It is therefore imperative to assess any claims of atypical growth strategies and, if they are present, to explore possible exceptions to the assumed “normal” life-history. Importantly, evidence of continued weight gain rather than skeletal growth is also informative about life-history. For example, it could shed light on different strategies between the sexes, such as in species in which males compete for mating opportunities using body size [29–31]. Investigating changes in body weight through life could also uncover declines in body weight with age that are potentially linked to senescence, as has been shown in several mammalian species [32–35]. This would allow for comparison with evidence of survival and reproductive senescence documented in many species [36–38].

Elephants have repeatedly been reported to indeterminately grow [39, 40]. It is critical to investigate this, as elephants are especially useful for life history studies; there is relatively little life-history data on mammalian species with long protracted lives at present that are proposed to grow indeterminately, and few other long-lived species can be studied in such depth. Older research based on height claimed that male African elephants *Loxodonta africana* and both sexes of Asian elephants *Elephas maximus* experience a secondary growth spurt [41, 42]. Jarman [39] then used this information from Laws et al. [41] to suggest elephants actually show indeterminate growth, rather than additional growth. Lindeque and van Jaarsveld [40] reached a similar conclusion with their own African elephant dataset. These studies all showed that weight of males is greater with age, though, as previously discussed, this is unlikely to be evidence for indeterminate growth. Furthermore, the studies of Laws et al. [41] and Lindeque and van Jaarsveld [40] were cross-sectional, with no repeated measures for individuals, meaning results would be more affected by temporal variation in individuals, and the ageing technique used is now known to be inaccurate [43], so whether the conclusions drawn reflect an actual biological phenomenon is up for debate. Regardless, despite the lack of solid evidence, the idea that elephants might have indeterminate growth still persists in the literature [44, 45].

In this study, we use a mix of cross-sectional and semi-longitudinal data from a semi-captive Asian elephant population in Myanmar to examine growth patterns of a long-lived mammal with slow life history in greater depth than previous work. The hypothesis that male elephants continue to grow throughout life whilst females reach a growth asymptote around the age of sexual maturity will be investigated. Specifically, we will define cross sectional and mixed longitudinal growth curves for the shoulder height (a measure of skeletal growth) and total body weight (a measure of body size) of 195 males and 255 females across the age range of 0–71 years using data from animals with known age and reproductive history, using subsets including animals of known age only and also including wild-caught animals with estimated ages. We then compare curves between males and females and assess the point at which skeletal growth cessation occurs in the two sexes and how this relates to adult changes in weight. The implications of these sex-specific strategies in relation to life history and reproduction and wildlife management are discussed.

## Results

### Growth curves

**Fig. 1:**
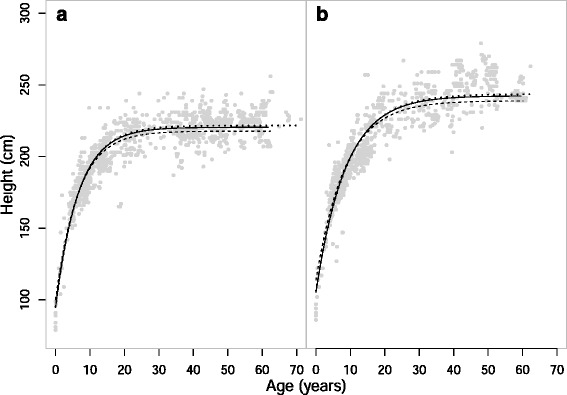
Height growth curves of **a**) females and **b**) males. Solid curves are derived from average measurements of captive-born elephants (*n* = 170 and 159); dotted curves are derived from average measurements of all elephants, of both wild- and captive-birth origin (*n* = 240 and 189); Dashed curves are derived from all captive-born elephants, including historic height data, and take ID into account (*n* = 22 and 26). Points are from all the elephants of that sex

**Fig. 2:**
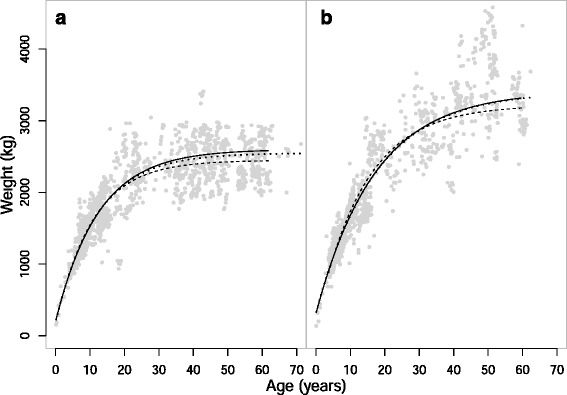
Weight growth curves of **a**) females and **b**) males. Solid curves are derived from average measurements of captive-born elephants (*n* = 172 and 159); dotted curves are derived from average measurements of all elephants, of both wild- and captive-birth origin (*n* = 243 and 188); dashed curves are derived from all captive-born elephants, including historic weight data, and take ID into account (*n* = 25 and 20). Points are from all the elephants of that sex

In order to investigate the question of whether Asian elephants exhibit indeterminate growth, we produced a total of twelve growth curves based on the three approaches discussed in the methods: cross-sectional with captive-born only, cross-sectional with all elephants regardless of birth-origin, and a longitudinal approach with captive-born only (Tables 1 and 2). There were three height curves and three weight curves for both males and females. Of the possible growth curve functions (Gompertz, von Bertalanffy and 3-parameter logistic), the one proposed by von Bertalanffy fitted each subset of data best, according to the coefficients of determination (see Additional file 1: Table S1), and visual assessment of fits (Additional file 1: Figures S1 and S2). The curves using the three different subsets of the data produced very similar results (Figs. 1 and 2), indicating the cross sectional curves are representative of individual growth curves.

**Table 1:** Parameters for height growth curves

Curve	*n*	*H∞* (cm)	*K*	*t*_*0*_
Averaged female, captive only	170	220	0.020	−1.93
Averaged female, wild and captive	240	222	0.018	−1.96
Longitudinal female, captive and historic	22	218	0.017	−1.89
Averaged male, captive only	159	243	0.006	−2.20
Averaged male, wild and captive	189	244	0.005	−2.23
Longitudinal male, captive and historic	26	240	0.003	−2.17

Von Bertalanffy curve parameters: *h*_*t*_ = *H∞*(1-e(−*K*(*t*-*t*_*0*_))), where *h*_t_ is the height of an individual at each age, *H∞* is the asymptotic (final) height of the population, *K* is the yearly growth rate, and *t*_*0*_ is the theoretical age at which height will be zero. Whilst the asymptotes of the curves are not statistically reached within the age range of the sampled elephants (0–71), this is a likely result of a lack of a fully longitudinal dataset that would span the entire lives of a large number of elephants

**Table 2:** Parameters for weight growth curves

Curve	*n*	*W∞* (cm)	*K*	*t*_*0*_
Averaged female, captive only	172	2599	0.37	−2.52
Averaged female, wild and captive	243	2548	0.40	−2.47
Longitudinal female, captive and historic	25	2498	0.49	−2.40
Averaged male, captive only	159	3412	0.20	−2.84
Averaged male, wild and captive	188	3407	0.19	−2.85
Longitudinal male, captive and historic	20	3238	0.44	−2.65

Von Bertalanffy: curve parameters *w*_*t*_ = *W∞*(1-e(−*K*(*t*-*t*_*0*_))), where *w*_t_ is the weight of an individual at age *t*, *W∞* is the asymptotic weight of the population, *K* is the growth rate, and *t*_*0*_ is the theoretical age at which weight will be zero. As with height, no formal statistical asymptotes of the curves were reached for weight in either sex (Table 1), likely for the same reason as for height: a lack of a fully longitudinal dataset

#### Height

The growth curves that best fit the data indicate growth of shoulder height of both male and female Asian elephants slows considerably after sexual maturity (around age 15) (Fig. 1), as expected for determinate growth. For females, 95 % of growth is completed around the age of sexual maturity, between 15–16 years old. For males 95 % of growth is completed slightly later, at around 21, although 90 % of their final height is achieved by age 15. At age 5, the age of weaning/independence, females are 73 % of their final height and males roughly 69 %; growth is most rapid during infancy (based on the steepness of the curve) and there is no evidence for an adolescent growth spurt. It must be noted that these values are percentages of the asymptote of the population, meaning that different individuals may reach this stage slightly earlier or slightly later than the average ages given. The asymptotes themselves represent the values that the average individual would achieve if they were to get old enough, meaning that in some cases individuals never reach the asymptotic height and that the asymptotic size predicted by the models could be higher than the adult height of the elephants if it were to be reached at an age no elephants are likely to attain. The final shoulder height of females predicted from these growth curves ranged between 218 cm and 222 cm (depending on the sample analysed; mean = 220 cm), with the captive-born only, cross-sectional curve having an asymptote of 220 cm. For males the range was similar, from 240 cm to 244 cm (mean = 242.3 cm), with the cross-sectional, captive-born only asymptote being just above the mean, at 243 cm. A full summary of the growth curve parameters can be found in Table 1.

#### Weight

The growth curves that best fit the data indicate that female weight gain is rapid up until peak fecundity (age 19 years), increasing from 38 % of final weight at age 5 to ~80 % by age 19. Weight gain then slows considerably, with 95 % of final weight attained by around 35 years. As with height, this is 95 % of the asymptote of the population. The pattern is also characteristic of determinate growth. Weight in males, however, continues to increase throughout life, though the curves do tend towards an asymptote, 95 % of which is reached by about 50 years. Unlike females, weight increase does not appear to slow considerably with age in males (Fig. 2), with weight at 19 only 70 % of final weight. Neither sex shows any evidence of declining body weight with age, for example the mean weight at the age of peak reproduction in females is ~2080 kg in contrast to ~2557 kg at age 50 when reproductive rates decline [37].

Female weight asymptotes ranged between 2498 kg and 2599 kg (depending on the sample analysed; mean = 2548 kg), and was highest for the captive-born only cross-sectional curve. The asymptotes for males represent the value that individuals would theoretically reach if they were to get old enough. The asymptote values were between 3238 kg and 3412 kg (mean = 3352 kg), with the cross-sectional captive-born curve highest at 3412 kg. However, as most males don’t achieve the age required to reach the weight asymptote in this population, the maximum weight a male can expect to reach in its lifetime ranges between 3178 kg and 3324 kg (mean = 3273 kg). For a full summary of parameters, see Table 2.

## Discussion

Indeterminate growth is near ubiquitous amongst invertebrates and fish, but it is not known if many other vertebrates follow this growth pattern. As some research areas and conservation measures rely heavily upon the assumptions of various life-history strategies, and as the growth strategy of a species is key to other aspects of its life history, it is of vital importance that reports of atypical growth are investigated. Our study has shown that against earlier suggestions, Asian elephants do not grow indeterminately. Though males and females differ in height, the pattern of height growth is very similar in both sexes and clearly determinate. However, weight differs in its manner of increase in the sexes, with female weight levelling off soon after maximum height is reached and male weight continuing to increase until death. Whilst this does not represent indeterminate growth, it could underlie some of the confusion in characterising the growth of species with body size dimorphism. Such findings have relevance for elephant research and conservation, but also more generally for comparative studies of life history.

Mammalian growth has long been thought to be determinate [6, 7], though some candidate species for indeterminate growth have been suggested in the literature [11, 13, 39, 46]. If any mammals exhibit this atypical strategy, then comparative life-history research and conservation efforts could be affected. Our results show that in the elephant population studied there is no continued increase in height much beyond maturity, which would be an indicator of skeletal growth and, therefore, indeterminate growth; it is clear that Asian elephants adhere to the typical mammalian growth strategy of determinate growth. It is unlikely that they violate traditional life-history assumptions in the manner of concepts like negative senescence [5]. However, this is only the case if the body weight increases that males experience are not linked to decreases in mortality. It is clear that links between body size and mortality in males represents an opportunity for further investigation into male ageing and life history strategies. Confirming that the growth of elephants is determinate allows them to be used in studies without the previous uncertainty surrounding their growth affecting any conclusions drawn, as has occurred in the literature [44]. There was also no evidence in this study to suggest secondary growth spurts in either males or females, as had been previously proposed by Sukumar et al. [42] for Asian elephants. The suggestion from Laws et al. [41] that male African elephants show this was dismissed in earlier literature as an artefact of inaccurate ageing [47]. However, this counter-argument does not apply to Sukumar et al. [42], as elephants were of known age. The elephants in this study were considered to have a growth spurt on the basis of some of their measured heights being greater than the asymptotes of the growth curves, rather than any clear pattern in the data.

The advantage of an increased weight for an increased dominance rank amongst males has been seen in species with sexual dimorphism and aggressive competition between males, such as red deer *Cervus elaphus* [29], Alpine ibex *Capra ibex* [30] and fallow deer *Dama dama* [18]. The weight curves presented here show a continuous, post-maturity increase in weight for males, whilst females asymptote much like for height; in the Asian elephant, gain in weight may therefore be related to the reproductive strategies of the sexes. Female elephants reach peak fecundity age 19 years old [36], by which time cessation of growth and weight gain is nearly complete; investment is likely switched to reproduction and increasing infant survival. Female reproductive rates decline more steeply from the age of 50 [37], but there is no associated decline in body weight in this study. Males, however, compete in aggressive interactions [48], with it being unlikely that bulls mate when they are low in the social hierarchy [49]. It is likely that mass plays a role in these interactions, as they are driven by a hormone-mediated condition known as musth, during which a male’s weight is greatly reduced [50]. Continuing to increase in weight throughout life would allow an individual to be in good condition for these competitive interactions at the onset of each musth, and this is seen in African elephants, where older males in musth are dominant over younger males [44]. Age at peak reproduction is unknown in male Asian elephants, but in African elephants the males reach peak reproduction much later in life [44], consistent with the idea that mass aids in dominance struggles. We find no evidence for a decline in body weight with increasing age in this study, although this has been documented in some other mammalian species [32–35]. One possibility is that low body weight individuals do not survive to advanced ages. A longitudinal study could further investigate this possibility.

Asian elephants are endangered [51], and neither the wild nor the captive Myanmar populations are self-sustaining [52], so if weight does have a strong influence on male elephant mating success, then conservation efforts ideally should be directed towards keeping enough elephants alive until such a time that they are heavy enough to be able to win dominance interactions and attain matings. This would keep genetic diversity within the population as high as possible. However, we first need to evaluate how important weight is for a male’s reproductive success by investigating dominance hierarchies and mating success through tracking and genetic paternity testing. Increasing weight with age in male Asian elephants was not found previously [42, 53], and females have also been said to increase in weight with age [53]. However, it must be noted that this conclusion was drawn from a study where curves were cross-sectional in the same manner of some of the African elephant studies [40, 41], so variation within individuals was not taken into account as it was in the cross-sectional curves presented in this work.

## Conclusions

In conclusion, this study on a large, known-age population of Asian elephants confirms that the species follows a pattern of determinate growth, which had previously been disputed in the literature. We show mature males and females differ in height, but follow the same pattern of growth. We also found that weight increases throughout life in males and levels off in females shortly after cessation of growth in height, highlighting the importance of measuring both parameters in mammals. In long-lived species with late ages at first reproduction, long-term weight gain may be closely linked to reproductive strategy, though little is currently known of the social structure of males or their reproductive success, making it difficult to assess the influence of weight in dominance interactions and mating success. Studies involving tracking of males and paternity testing may go some way to addressing these gaps.

## Methods

### Study population

Asian elephants are an endangered species [51] distributed discontinuously across Southeast Asia and the Indian sub-continent. The Union of Myanmar has the second largest wild population of Asian elephants numbering up to five thousand individuals [54, 55], and the largest captive one, also of around five thousand elephants [54]. Of this captive population, 2700 are government owned through the Myanma Timber Enterprise and are used for sustainable logging [52]. Not all the elephants are born in captivity; historically, elephants have been captured from the wild to supplement the population [56] and these comprise almost half of the elephants alive today.

The logging elephants are classified as semi-captive, as there is no provisioning or selective breeding [50]. The animals are never culled, so there cannot be selective survival based on size that is human-driven (although there can of course be due to natural causes of death). The elephants are released into the forest at night for up to fourteen hours to forage at will and mate unsupervised [56], where both wild and captive bulls have access to oestrus females. Elephants work between mid-June and mid-February with a rest period during October [57]. This working season is timed to coincide with the monsoon (July-October) and cool (November-February) seasons, so that no work is done during the intense heat of the dry season (March-June), when temperature-related mortality is highest [58]. Each elephant has a maximum tonnage that they move each year, and have strict limits to daily and weekly work that cannot be exceeded – in 2010 these limits were set to no more than eight hours a day, with a break at noon, and no more than five days of work in a week [36].

Elephants are born weighing approximately 100 kg and standing at around 1 m. Following birth calves remain at the heel of the mother, being exclusively dependent on lactation until around 6 months and continuing to suckle until the age of around 3–5 years [59]. Calves become independent at the age of 5 when they are separated from the mother and trained. From age 5 years until 17, elephants are only used for light work, becoming part of the true working population after this. Alongside this the elephants become reproductively mature, with bulls experiencing first musth at around the age of 15 and females beginning oestrus cycles at around 10–12 years [60]. Whilst the timing of sexual maturity is similar in males and females, the first recorded reproduction by a female is at age 5 and the age at peak fecundity is 19 years, followed by a slow decline [36]. In contrast male Asian elephants are unlikely to reproduce until around the age of 25 [60]. Myanmar timber elephants are defined as adults at the age of 17 and are able to drag logs and engage in heavy work, continuing up until around 55 years old, at which point they are retired from work [50]. Asian elephants can live for up to 80 years, and can therefore spend decades in retirement [37]. Their reproductive careers can also span many decades [61], with reproduction declining more steeply from age 50, but mothers continuing to give birth into their 60’s [50]. Mothers are given time off work from mid-pregnancy (at around 11 months) through to a year after birth [56], and are then given lighter work up until the infant is weaned, at which point the mother returns to heavier work.

Each elephant is marked with its own unique identification (ID) number, and important life-history information is recorded in log books as dictated by law [50, 62], and includes, but is not limited to, birth origin (captive or wild), mother ID, health information and dates of birth and death. Age of wild-born individuals is assessed visually at capture to the nearest year using skin pigmentation, ear tears and folding, facial concavity and size (for younger individuals) [53, 63]. These assessment measurements have shown to be particularly accurate for young elephants [64]. Any body measurements taken by vets at their two weekly health checks are also included in these log books. This population, containing a large number of known individuals of both sexes for whom data have been collected over time, is ideal for testing the growth patterns of both sexes over the entire lifespan.

### Data selection

Height and weight were recorded in five logging camps in Myanmar: Pyinmana, Monywa, East Katha, West Katha and Kawlin. Weight was measured using EziWeigh 3000 scales, and was recorded to the nearest 1 kg, whilst the other measurements were taken with tape measures, and in most cases recorded to the nearest inch. At some camps, measurements were taken in centimetres. As a result, all measurements were converted into centimetres during collation of data, but are only correct to the nearest multiple of 2.54 cm (i.e., 1 in.). In total 450 elephants aged between 0 and 71 were included in this study, with each individual having at least one body measurement. Of these, 255 were female and 195 were male.

In Pyinmana (*n* = 72), individuals were measured monthly between December 2011 and October 2012. Elephants were recorded cross-sectionally just once in Monywa in 2012 (*n* = 74). In East Katha (*n* = 73), data were collected monthly from December 2012 until the end of 2013. In West Katha (*n* = 58), monthly measurements have been taken since June 2012, with data up until March 2015 available. Kawlin (*n* = 173) data have been collected monthly since November/December 2012, with records up until April 2015 available. However, for camps where monthly measurements had been taken, some months did not have data. Some historic height measurements from the logbooks from Kawlin elephants, taken sporadically since 1972, were also available for use.

### Statistical analyses

All analyses were conducted using *R* 3.1.3 [65]. To test for the presence of indeterminate growth in elephants, as measured by both their weight and height gain across age, we implemented non-linear least squares models for each sex using the *nls* function, with a response variable of weight or height. We applied three commonly used self-starting growth functions as the explanatory variable to obtain the best fit: Gompertz, three-parameter logistic and von Bertalanffy [17, 43]. The growth function producing the best fit was selected by the coefficient of determination, with the highest value providing the best fit. We used age in years as the input parameter. In all models we calculated i) age at which 95 % of asymptotic weight/height was reached, as a marker of age at growth cessation ii) proportion of asymptotic weight/height achieved by age at weaning and independence (defined as age 5), to investigate how much growth had taken place up to this important life history transition point, ii) proportion of asymptotic weight/height achieved by age at sexual maturity (defined as age 15), as an indicator of whether growth continued after sexual maturity and could therefore be defined as indeterminate and iii) the age at which growth was most rapid, to investigate the overall pattern of growth.

We tested each of the three growth functions in three different subsets of our dataset as follows. First, we implemented them in captive-born individuals of known age (*n* = 348). The average measurements and age of individuals from the monthly measures available for them between 2011 and 2015 were used for this analysis, therefore historic data for individuals were also excluded at this stage as this could give an unrepresentative size at the average age. We produced separate growth curves for males and females, for both height and weight as they may have different growth patterns. We then produced additional sets of growth curves that included the wild-born and unknown origin individuals (*n* = 104) alongside the captive-born individuals using the same self-starting growth functions, in order to compare the growth curves to those in which all individuals have accurate age data.

Finally, we made a third set of growth curves that took into account within individual variation and longitudinal measurements. Only captive elephants with a minimum of five separate measurements of data (mean number of measures per individual = 7.9) were selected for this analysis (*n* = 220 measurements from 48 individuals for height and 45 individuals for weight), so as to have enough longitudinal measurements without reducing the sample size substantially. We also included the historic measurements from Kawlin, providing growth data over a much longer period (up to 43 years); as many of these measurements were taken yearly for over up to several decades. For these curves, there was not complete growth data across the entire lifespan of individuals to justify a fully longitudinal model and the polynomial approach used for mixed longitudinal studies is not suited to data that has an asymptote [66]. Instead we applied another approach for longitudinal data using the *groupedData*, *nlsList* and *nlme* functions from the package *nlme* version 3.1–120 [67]. We specified ID as a grouping factor in the grouping call, and in the non-linear mixed model the function *SSasympOff* was used in order to allow data to originate through points other than 0. We set the asymptote as a random effect, allowing it to vary by ID to account for between-individual differences.

### Availability of supporting data

The data set supporting the results of this article will be made available in the Dryad repository.

We also supply one Additional File including a table and two figures (Additional file 1).

### Ethics approval

The data collection supporting this study was approved by the University of Sheffield Ethics Committee.

## Additional file

Additional file 1: Table S1. Coefficients of determination for growth curves generated with the growth functions Gompertz, 3-Parameter Logistic and von Bertalanffy. Bolded values show the function that provides the best fitting growth curve. **Figure S1.** Height curves for a) females and b) males, derived from average measurements of captive-born individuals (*n* = 170 and 159). Each line indicates a different growth function: von Bertalanffy represented by a solid line, 3-parameter logistic by dashes, and Gompertz by a dotted line. **Figure S2.** Weight curves for a) females and b) males, derived from average measurements of captive-born individuals (*n* = 172 and 159). Each line indicates a different growth function: von Bertalanffy represented by a solid line, 3-parameter logistic by dashes, and Gompertz by a dotted line. (PDF 483 kb)

## References

[1] Vinicius L, Mumby HS (2013). Comparative analysis of animal growth: A primate continuum revealed by a new dimensionless growth rate coefficient. Evolution.

[2] Stearns SC (1992). The Evolution of Life Histories.

[3] Charnov EL (1993). Life History Invariants.

[4] Sgrò CM, Partridge L (1999). A delayed wave of death from reproduction in Drosophila. Science.

[5] Vaupel JW, Baudisch A, Dölling M, Roach DA, Gampe J (2004). The case for negative senescence. Theor Popul Biol.

[6] Sebens KP (1987). The Ecology of Indeterminate Growth in Animals. Annu Rev Ecol Syst.

[7] Charnov EL, Turner TF, Winemiller KO (2001). Reproductive constraints and the evolution of life histories with indeterminate growth. Proc Natl Acad Sci U S A.

[8] Congdon JD, Gibbons JW, Brooks RJ, Rollinson N, Tsaliagos RN (2013). Indeterminate growth in long-lived freshwater turtles as a component of individual fitness. Evol Ecol.

[9] Shine R, Charnov EL (1992). Patterns of survival, growth, and maturation in snakes and lizards. Am Nat.

[10] Kratochvíl L, Frynta D (2002). Body size, male combat and the evolution of sexual dimorphism in eublepharid geckos (Squamata: Eublepharidae). Biol J Linn Soc.

[11] Leigh SR, Shea BT (1995). Ontogeny and the evolution of adult body size dimorphism in apes. Am J Primatol.

[12] Trites AW, Bigg MA (1996). Physical growth of northern fur seals (Callorhinus ursinus): seasonal fluctuations and migratory influences. J Zool Soc London.

[13] Deutsch CJ, Crocker DE, Costa DP, Le Boeuf BJ, Laws RM (1994). Sex- and age-related variation in reproductive effort of Northern elephant seals. Elephant seals: Population ecology, behavior, and physiology.

[14] Sibly R, Calow P, Nichols N (1985). Are patterns of growth adaptive?. J Theor Biol.

[15] Ernsting G, Zonneveld C, Isaaks JA, Kroon A (1993). Size at maturity and patterns of growth and reproduction in an insect with indeterminate growth. Oikos.

[16] Heino M, Kaitala V (1999). Evolution of resource allocation between growth and reproduction in animals with indeterminate growth. J Evol Biol.

[17] Karkach AS: Trajectories and models of individual growth. Demogr Res. 2006;347-400 15.

[18] McElligott AG, Gammell MP, Harty HC, Paini DR, Murphy DT, Walsh JT, Hayden TJ: Sexual size dimorphism in fallow deer (Dama dama): do larger, heavier males gain greater mating success? 2001. Behavioral Ecology and Sociobiology. 49:266–72.

[19] Schwitzer C, Kaumanns W (2001). Body weights of ruffed lemurs (Varecia variegata) in European zoos with reference to the problem of obesity. Zoo Biol.

[20] Videan EN, Fritz J, Murphy J (2007). Development of guidelines for assessing obesity in captive chimpanzees (Pan troglodytes). Zoo Biol.

[21] Ely JJ, Zavaskis T, Lammey ML (2013). Hypertension Increases With Aging and Obesity in Chimpanzees (Pan troglodytes). Zoo Biol.

[22] Kuhar CW, Fuller GA, Dennis PM (2013). A survey of diabetes prevalence in zoo-housed primates. Zoo Biol.

[23] Less EH, Bergl R, Ball R, Dennis PM, Kuhar CW, Lavin SR, Raghanti MA, Wensvoort J, Willis MA, Lukas KE (2013). Implementing a low-starch biscuit-free diet in zoo gorillas: The impact on behavior. Zoo Biol.

[24] Plavcan JM (2012). Sexual Size Dimorphism, Canine Dimorphism, and Male-Male Competition in Primates: Where Do Humans Fit In?. Hum Nat.

[25] Hamidan N, Britton JR (2015). Age and growth rates of the critically endangered fish Garra ghorensis can inform their conservation management. Aquat Conserv Mar Freshw Ecosyst.

[26] Arlinghaus R, Matsumura S, Dieckmann U (2010). The conservation and fishery benefits of protecting large pike (Esox lucius L.) by harvest regulations in recreational fishing. Biol Conserv.

[27] Becker CG, Loyola RD, Haddad CFB, Zamudio KR (2010). Integrating species life-history traits and patterns of deforestation in amphibian conservation planning. Divers Distrib.

[28] Ochwada-Doyle F, Roberts D, Gray C, Barnes L, Haddy J, Fearman J (2012). Characterizing the biological traits and life history of Acanthopagrus (Sparidae) hybrid complexes: Implications for conservation and management. J Fish Biol.

[29] Clutton-Brock TH, Albon SD, Gibson RM, Guinness FE (1979). The logical stag: Adaptive aspects of fighting in red deer (Cervus elaphus L.). Anim Behav.

[30] Bergeron P, Grignolio S, Apollonio M, Shipley B, Festa-Bianchet M (2010). Secondary sexual characters signal fighting ability and determine social rank in Alpine ibex (Capra ibex). Behav Ecol Sociobiol.

[31] Hunt J, Breuker CJ, Sadowski JA, Moore AJ (2009). Male-male competition, female mate choice and their interaction: Determining total sexual selection. J Evol Biol.

[32] Monteith KL, Bleich VC, Stephenson TR, Pierce BM, Conner MM, Kie JG, Bowyer RT: Life-history characteristics of mule deer: Effects of nutrition in a variable environment. Wildl Monogr 2014;186:1–62.

[33] Nussey DH, Coulson T, Delorme D, Clutton-Brock TH, Pemberton JM, Festa-Bianchet M, Gaillard JM (2011). Patterns of body mass senescence and selective disappearance differ among three species of free-living ungulates. Ecology.

[34] Hämäläinen A, Dammhahn M, Aujard F, Eberle M, Hardy I, Peter M, Perret M, Schliehe-diecks S, Kraus C, B PRS, Kappeler PM: Senescence or selective disappearance? Age trajectories of body mass in wild and captive populations of a small-bodied primate. Proc R Soc B 2014, 281:20140830. 10.1098/rspb.2014.0830 PMC4132673 25100693

[35] Tafani M, Cohas A, Bonenfant C, Gaillard JM, Lardy S, Allainé D (2013). Sex-specific senescence in body mass of a monogamous and monomorphic mammal: The case of Alpine marmots. Oecologia.

[36] Hayward AD, Mar KU, Lahdenperä M, Lummaa V (2014). Early reproductive investment, senescence and lifetime reproductive success in female Asian elephants. J Evol Biol.

[37] Lahdenperä M, Mar KU, Lummaa V (2014). Reproductive cessation and post-reproductive lifespan in Asian elephants and pre-industrial humans. Front Zool.

[38] Mumby HS, Mar KU, Hayward AD, Htut W, Htut-Aung Y, Lummaa V: Elephants born in the high stress season age faster. Sci Rep. 2015, In Press. 10.1038/srep13946 PMC4568471 26365592

[39] Jarman P (1983). Mating system and sexual dimorphism in large, terrestrial, mammalian herbivores. Biol Rev.

[40] Lindeque M, van Jaarsveld AS (1993). Post-natal growth of elephants Loxodonta africana in Etosha National Park, Namibia. J Zool.

[41] Laws RM, Parker ISC, Johnstone RCB (1975). Elephants and Their Habitats.

[42] Sukumar R, Joshi NV, Krishnamurthy V (1988). Growth in the Asian elephant. Proc Indian Acad Sci Animal Sci.

[43] Shrader AM, Ferreira SM, McElveen ME, Lee PC, Moss CJ, van Aarde RJ (2006). Growth and age determination of African savanna elephants. J Zool.

[44] Hollister-Smith JA, Poole JH, Archie EA, Vance EA, Georgiadis NJ, Moss CJ, Alberts SC (2007). Age, musth and paternity success in wild male African elephants, Loxodonta africana. Anim Behav.

[45] Chiyo PI, Lee PC, Moss CJ, Archie EA, Hollister-Smith JA, Alberts SC (2011). No risk, no gain: Effects of crop raiding and genetic diversity on body size in male elephants. Behav Ecol.

[46] Maynes GM (1976). Growth of parma wallaby, Macropus parma Waterhouse. Aust J Zool.

[47] Hanks J (1972). Growth of the African elephant (Loxodonta africana). East African Wildl J.

[48] Lincoln GA, Ratnasooriya WD (1996). Testosterone secretion, musth behaviour and social dominance in captive male Asian elephants living near the equator. J Reprod Fertil.

[49] Sukumar R (1989). The Asian Elephant: Ecology and Management.

[50] Mar KU: The demography and life history startegies of timber elephants in MyanmarKhyne U Mar Thesis submitted to the University College London. Changes 2007;5:156.

[51] Elephas maximus [www.iucnredlist.org]. 10th June 2015

[52] Leimgruber P, Senior B, Uga, Aung M, Songer MA, Mueller T, Wemmer C, Ballou JD: Modeling population viability of captive elephants in Myanmar (Burma): Implications for wild populations. Anim Conserv. 2008, 11:198–205.

[53] Kurt F, Kumarasinghe C. Remarks on body growth and phenotypes in Asian elephant Elephas maximus. Acta Theriol. 1998:135–53.

[54] Sukumar R (2006). A brief review of the status, distribution and biology of wild Asian elephants Elephas maximus. Int Zoo Yearb.

[55] Leimgruber P, Min Oo Z, Aung M, Kelly DS, Wemmer C, Senior B, Songer M (2011). Current status of Asian elephants in Myanmar. Gajah 35.

[56] Gale T (1974). Burmese Timber Elephants.

[57] Mumby HS, Courtiol A, Mar KU, Lummaa V (2013). Birth seasonality and calf mortality in a large population of Asian elephants. Ecol Evol.

[58] Mumby HS, Courtiol A, Mar KU, Lummaa V (2013). Climatic variation and age-specific survival in Asian elephants from Myanmar. Ecology.

[59] Mar KU, Lahdenperä M, Lummaa V (2012). Causes and correlates of calf mortality in captive asian elephants (elephas maximus). PLoS One.

[60] Hildebrandt TB, Göritz F, Hermes R, Reid C, Dehnhard M, Brown JL (2006). Aspects of the reproductive biology and breeding management of Asian and African elephants. Int Zoo Yearb.

[61] Robinson MR, Mar KU, Lummaa V (2012). Senescence and age-specific trade-offs between reproduction and survival in female Asian elephants. Ecol Lett.

[62] Zaw K (1997). Utilization of Elephants in Timber Harvesting in Myanmar. Gajah.

[63] Arivazhagan C, Sukumar R (2008). Constructing age structures of Asian elephant populations: A comparison of two field methods of age estimation. Gajah.

[64] De Silva S, Elizabeth Webber C, Weerathunga US, Pushpakumara TV, Weerakoon DK, Wittemyer G (2013). Demographic variables for wild Asian elephants using longitudinal observations. PLoS One.

[65] R Core Team: R. A language and environment for statistical computing. 2015. http://www.r-project.org/.

[66] Huggins RM, Loesch DZ (1998). On the analysis of mixed longitudinal growth data. Biometrics.

[67] Pinheiro J, Bates D, DebRoy S, Sarkar D, R Core Team: nlme: Linear and nonlinear mixed effects models. 2015. http://cran.r-project.org/package=nlme.

