# Correction to: Distinguishing between determinate and indeterminate growth in a long-lived mammal

**DOI:** 10.1186/s12862-021-01951-1

**Published:** 2021-12-06

**Authors:** Hannah S. Mumby, Simon N. Chapman, Jennie A. H. Crawley, Khyne U. Mar, Win Htut, Aung Thura Soe, Htoo Htoo Aung, Virpi Lummaa

**Affiliations:** 1 Department of Animal and Plant Sciences, University of Sheffield, Western Bank, Sheffield, S10 2TN UK; 2 Ministry of Environmental Conservation and Forestry, Myanmar Timber Enterprise, Yangon, Myanmar

## Correction to: BMC Evol Biol (2015) 15:1 https://doi.org/10.1186/s12862-015-0487-x

Following the publication of the original article [1], we were notified of the following corrections: in both Tables 1 and 2 (height and weight), the values for K should be the exponent of the values in the t_0_ column, and the values in t_0_ should be the log of the values in K. This does not affect the curves, as the authors used a predict function.

Correct Tables 1 and 2 can be found in this erratum:

**Table 1:** Parameters for height growth curves

Curve	*n*	*H∞* (cm)	*K*	*t*_*0*_
Averaged female, captive only	170	220	0.145	−3.91
Averaged female, wild and captive	240	222	0.141	−4.02
Longitudinal female, captive and historic	22	218	0.151	−4.08
Averaged male, captive only	159	243	0.111	−5.12
Averaged male, wild and captive	189	244	0.108	−5.30
Longitudinal male, captive and historic	26	240	0.114	−5.81

**Table 2:** Parameters for weight growth curves

Curve	*n*	*W∞* (kg)	*K*	*t*_*0*_
Averaged female, captive only	172	2599	0.081	−0.99
Averaged female, wild and captive	243	2548	0.085	−0.92
Longitudinal female, captive and historic	25	2498	0.091	−0.71
Averaged male, captive only	159	3412	0.058	−1.61
Averaged male, wild and captive	188	3407	0.058	−1.66
Longitudinal male, captive and historic	20	3238	0.071	−0.82
