# Supplementary material for: Distinguishing between determinate and indeterminate growth in a long-lived mammal
Source: BMC Evol Biol. 2015 Oct 14;15:214. doi: 10.1186/s12862-015-0487-x (PMC4604763; doi:10.1186/s12862-015-0487-x)

Table S1. Coefficients of determination for growth curves generated with the growth functions Gompertz, 3-Parameter Logistic and von Bertalanffy. Bolded values show the function that provides the best fitting growth curve.

	Growth curve function coefficients of determination		
Growth Curve	Gompertz	von Bertalanffy	3-Parameter Logistic
Female (Height)	0.873	**0.881**	0.865
Female (Weight)	0.838	**0.842**	0.832
Male (Height)	0.896	**0.905**	0.888
Male (Weight)	0.881	**0.886**	0.873

Figure S1. Height curves for a) females and b) males, derived from average measurements of captive-born individuals (*n* = 170 and 159). Each line indicates a different growth function: von Bertalanffy represented by a solid line, 3-parameter logistic by dashes, and Gompertz by a dotted line.

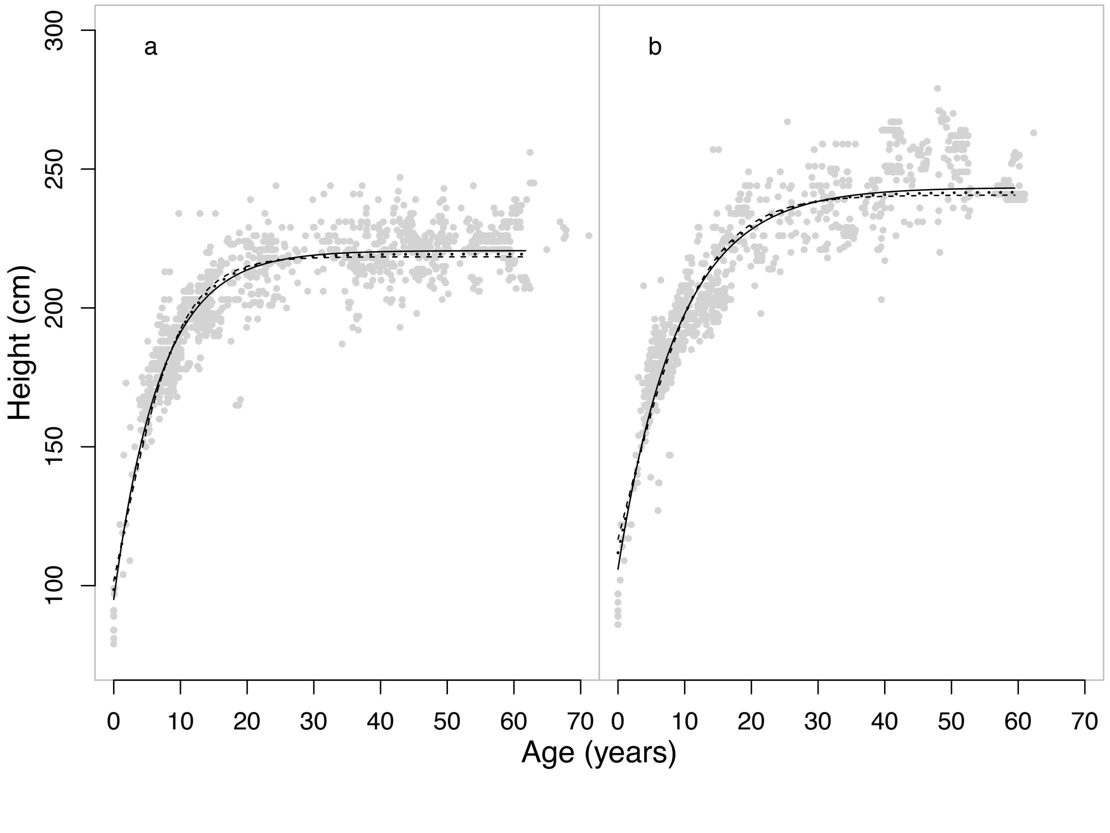

Figure S2. Weight curves for a) females and b) males, derived from average measurements of captive-born individuals (n = 172 and 159). Each line indicates a different growth function: von Bertalanffy represented by a solid line, 3-parameter logistic by dashes, and Gompertz by a dotted line.

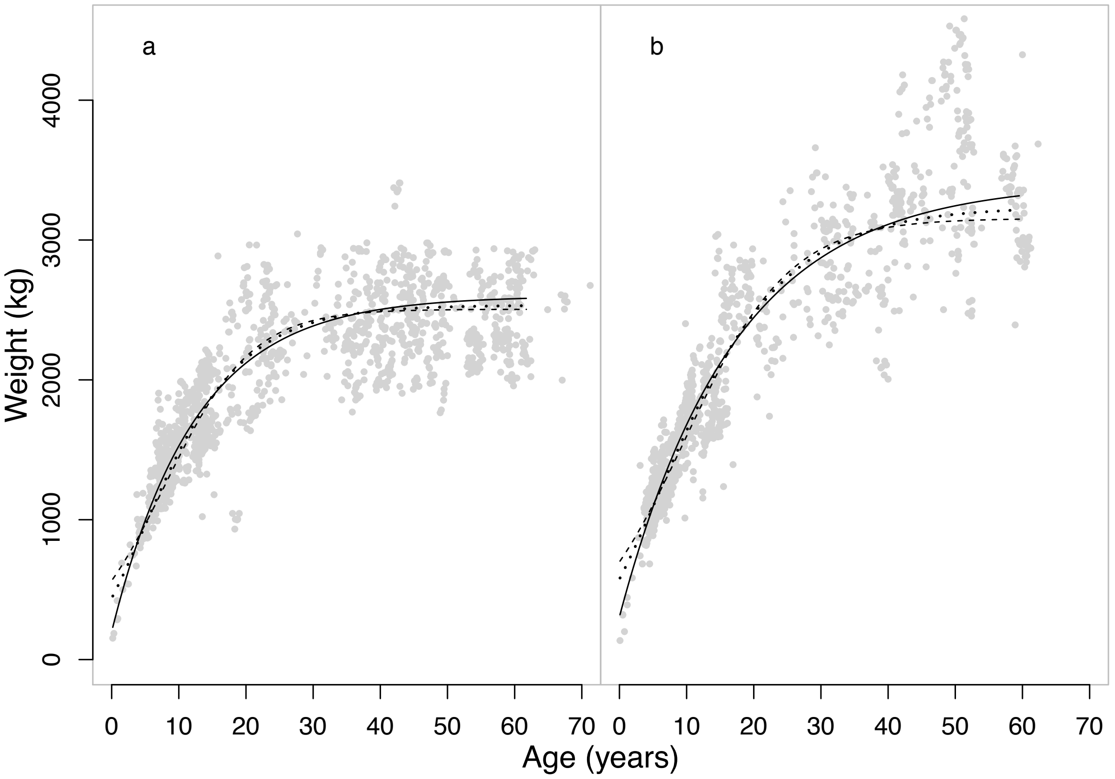

Supplement: Additional file 1: Table S1. — Coefficients of determination for growth curves generated with the growth functions Gompertz, 3-Parameter Logistic and von Bertalanffy. Bolded values show the function that provides the best fitting growth curve. Figure S1. Height curves for a) females and b) males, derived from average measurements of captive-born individuals (n = 170 and 159). Each line indicates a different growth function: von Bertalanffy represented by a solid line, 3-parameter logistic by dashes, and Gompertz by a dotted line. Figure S2. Weight curves for a) females and b) males, derived from average measurements of captive-born individuals (n = 172 and 159). Each line indicates a different growth function: von Bertalanffy represented by a solid line, 3-parameter logistic by dashes, and Gompertz by a dotted line. (PDF 483 kb) [file 12862_2015_487_MOESM1_ESM.pdf]
